# Atorvastatin reduces cerebral vasospasm and infarction after aneurysmal subarachnoid hemorrhage in elderly Chinese adults

**DOI:** 10.18632/aging.102788

**Published:** 2020-02-07

**Authors:** Junhui Chen, Mingchang Li, Xun Zhu, Lei Chen, Shuo Yang, Chunlei Zhang, Ting Wu, Xiaoyan Feng, Yuhai Wang, Qianxue Chen

**Affiliations:** 1 Department of Neurosurgery, Renmin Hospital of Wuhan University, Wuhan 430060, Hubei, China; 2 Department of Neurosurgery, Wuxi Clinical College of Anhui Medical University, 904th Hospital of Joint Logistic Support Force of PLA, Wuxi 214044, China; 3 Department of Cardiology, Wuxi Clinical College of Anhui Medical University, 904th Hospital of Joint Logistic Support Force of PLA, Wuxi 214044, China

**Keywords:** atorvastatin, subarachnoid hemorrhage, CVS, RCT

## Abstract

We explored whether acute atorvastatin treatment would improve clinical outcomes and reduce the incidence of cerebral vasospasm after aneurysmal subarachnoid hemorrhage in elderly Chinese adults. Patients (60 to 90 years old) were admitted to intensive care units after surgery to clip or embolize their aneurysms. We assessed 592 patients and assigned 159 to receive atorvastatin (20 mg/day) and 158 to receive placebo once daily for up to 14 days. The primary outcome was the Glasgow outcome scale at 6 months, and secondary outcomes were cerebral vasospasm, 30-days all-cause mortality, cerebral infarction, and delayed ischemic neurological deficit. The incidence of postoperative cerebral vasospasm (39.3% vs 56%, *P* =0.004) and cerebral infarction (18.7% vs 27.3%, *P*=0.027) were significantly lower in the atorvastatin group. The study did not detect benefits in the use of atorvastatin for 6 months clinical outcome or 30-day all-cause mortality, but it suggests that atorvastatin together with nimodipine can reduce cerebral vasospasm and cerebral infarction after subarachnoid hemorrhage.

## INTRODUCTION

Spontaneous subarachnoid hemorrhage (SAH) is the most common cerebral vascular disease, and 75% of SAHs are caused by rupture of an intracranial aneurysm [1–3]. The fatality rate is 40%, and many survivors have long-term neurological and cognitive impairment. SAH is still associated with mortality at one month for half of all patients, and another quarter is left disabled [4, 5]. Even though some patients survive the initial aneurysmal SAH (aSAH), several complications can contribute to poor outcome. One of the most important causes of resulting mortality and morbidity is cerebral vasospasm (CVS) and CVS- related ischemic infarcts causing delayed cerebral ischemia (DCI) [6]. After aSAH, CVS was observed on angiography in more than 70% of patients, and 18% to 56% of patients demonstrated secondary ischemia with clinical deterioration, which is significantly and independently associated with poor outcome [7, 8]. Current management guidelines just nimodipine only to relieve CVS, but the efficacy of this drug treatment is suboptimal [9].

Statins, 3-hydroxy-3-methylglutaryl coenzyme A (HMG-CoA) reductase inhibitors widely used in cardio-vascular medicine as cholesterol-lowering drugs, have been suggested to exert pleiotropic effects. Animal studies have shown that statins can reduce inflammation, protect vascular endothelial cells, improve brain edema and decrease platelet activation [10–12]. Our experimental evidence also indicates that atorvastatin may improve aSAH outcomes through its inhibition of AQP4 and ET-1 expression and protection of the autoregulation of cerebral vessels [11, 12]. Acute statin therapy, therefore, may present a promising candidate for SAH clinical treatment [13]. Unfortunately, a recent multicentre randomized phase 3 trial of simvastatin (STASH) did not reveal any value for long-term or short-term outcomes in aSAH patients [14]. The heterogeneous populations studies by race and age may obscure clinical benefits limited to certain populations. Our pre-experiment indicated that improved outcomes mostly occurred in older aSAH patients. We therefore explored whether acute atorvastatin treatment would reduce CVS, Vasospasm-related ischemic infarcts and improve clinical outcomes after aneurysmal SAH in a population of elderly Chinese adults.

## RESULTS

**Figure 1:**
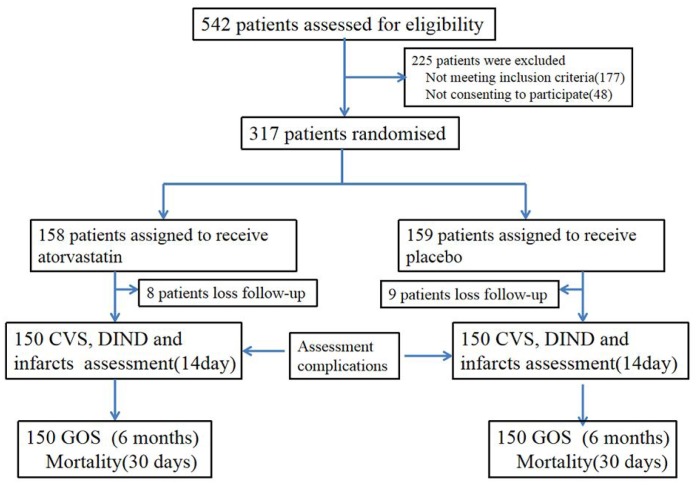
**Trial profile.** VS: cerebral vasospasm. DIND: delayed ischemic neurological deficit.

**Figure 2:**
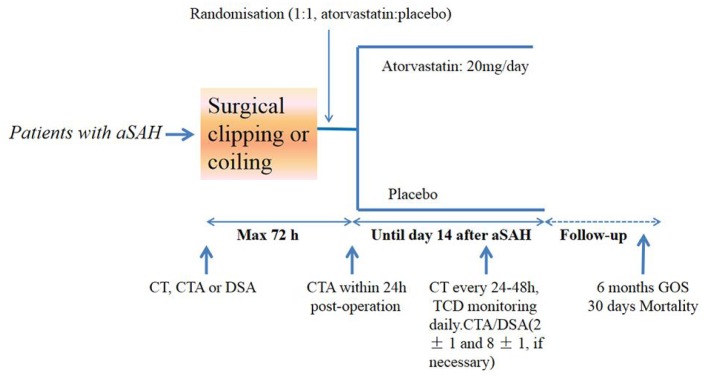
**Study design.** aSAH=aneurysmal subarachnoid hemorrhage. GOS= Glasgow outcome scale. TCD: Transcranial Doppler

Of 592 patients assessed between Oct 1, 2014, and Oct 1, 2017, 317 were randomly assigned to receive either placebo (n=159) or atorvastatin (n=158); 17 patients ended treatment or were lost to follow-up (Figure 1). There were no lapses in the blinding during the study period. There were no statistically significant differences in baseline characteristics between the atorvastatin and placebo groups (Table 1). All patients were included in the final intention-to-treat analyses (Figure 2). The final visit of the last randomized patient was on Mar 20, 2018. Outcome data were unavailable for one patient in the placebo group and two in the atorvastatin group at 6 months. For the GOS primary endpoint, 2 patients had data substituted in the atorvastatin group (one with GOS score 3; one with GOS score 4), and one patient had data substituted in the placebo group (with GOS score 4). No data were lost in the other assessments such as all-cause mortality at 30 days after aSAH, CVS, vasospasm-related new infarction and delayed ischemic neurological deficit (DIND) due to vasospasm within 2 weeks post-aSAH.

**Table 1:** Demographic and baseline characteristics of the study population in two group.

	**Placebo group**	**Atorvastatin group**
Number of patients		
Age	150	150
Mean ± SD	75.21±1.7	76.1±11.1
Gender		
Male	63(42%)	73(48.7%)
Female	87(58%)	77(51.3%)
History of hypertension		
Yes	51(34.0%)	48(32%)
No	99(66.0%)	102(68%)
Nicotine use		
Yes	35(23.3%)	40(26.7%)
No	115(76.7%)	110(73.3%)
Hunt-Hess grade		
I-III	122(81.3%)	113(75.3%)
IV	28(18.7%)	37(24.7%)
Aneurysm location		
Anterior circulation	143(95.3%)	139(92.7%)
Posterior Circulation	7(4.7%)	11(7.3%)
Aneurysm size		
<5mm	56(37.3%)	64(42.7%)
>5mm	94(62.7%)	86(57.3%)
Clot size		
Diffuse thick	78(52.0%)	67(44.7%)
Diffuse thin/localthick/local thin	72(48.0%)	83(55.3%)
Surgical procedure		
clipping	130(86.7%)	138(92.0%)
coiling	20(13.3%)	12(8.0%)

### Demographic and clinical data

The demographics and baseline characteristics of patients were similar between the atorvastatin and placebo groups (Table 1). Most of the patients were women with Hunt-Hess grades I to III subarachnoid hemorrhages. The mean treatment duration was 14 days (SD = 2) for the placebo-treated group and 13 days (SD = 2) for the atorvastatin-treated group. Oral and injected nimodipine were administered to all participants receiving atorvastatin and placebo, with no significant difference observed between the two.

### The primary endpoint-clinical outcomes

**Figure 3:**
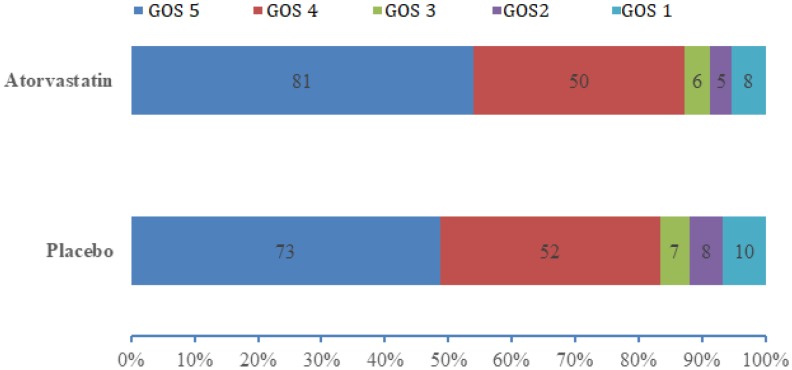
**Distributions of GOS score in the atorvastatin and placebo groups.** Data are number of patients with each GOS score. Tested with Mann-Whitney U test; *P*=0.393.

At 6 months, there were no significant differences in GOS classification between the two groups (Table 2 and Figure 3). A higher percentage of patients in the atorvastatin (54.4%, 81/150) group had good recovery compared with patients in the placebo (48.7%, 73/450) group, but statistical significance was not reached (*P*=0.36, 95% CI 0.72–1.12). Ten (6.6%) patients in the placebo group and 8 (5.3%) in the atorvastatin group died during the study.

**Table 2:** Comparison of primary endpoint-clinical outcomes between the two groups.

**Variable**	**Placebo group**	**Atorvastatin group**	***P* value**
Number of patients	150	150	
GOS			0.393
Good recovery	73(48.7%)	81(54%)	
Moderate disability	52(34.7%)	50(33.3%)	
Severe disability	7(4.7%)	6(4%)	
Vegetative state	8(5.3%)	5(3.4%)	
Dead	10(6.6%)	8(5.3%)	
30-day mortality	8(5.3%)	7(4.7%)	0.791

Data are presented as numbers (%) and were compared between groups using the Pearson Chi-square test and rank sum test.

**Figure 4:**
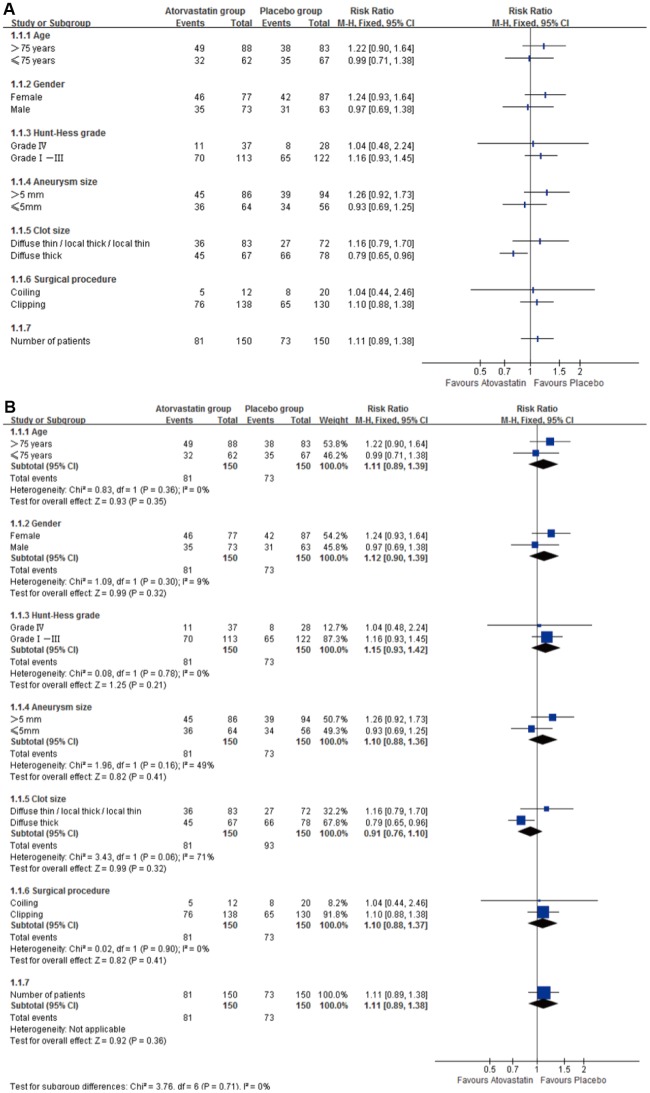
**Subgroup analyses for primary outcome Subgroup analyses for good outcome (GOS 5), RR=risk ratio.** (**A**) Subgroup analyses for good outcome (GOS 5) in age, Hunt-Hess, clot size and surgical procedure, gender and aneurysm size showed no difference between groups. (**B**) Heterogeneity test showed no heterogeneity in age, Hunt-Hess, clot size and surgical procedure subgroups (I^2^=0); gender (I^2^=9%) and aneurysm size (I2=28%) subgroups had no significant heterogeneity.

Subgroup analyses for a good outcome (GOS 5) showed no effect of gender, age (>75 or <75), aneurysm size, clot size, good or poor clinical condition at admission (Hunt-Hess grades), and different surgical procedure (Table 3 and Figure 4A). Also, heterogeneity test showed that no heterogeneity in age, Hunt-Hess, and surgical procedure subgroups (I^2^=0); gender (I^2^=9%) and aneurysm size (I^2^=28%) subgroups had no significant heterogeneity. Clot size had significant heterogeneity (I^2^=71%, Figure 4B).

**Table 3:** Date of subgroup analyses.

**Group**	**Placebo group**	**Atorvastatin group**
Number of patients	73/150	81/150
Age		
≤75 years	35/67	32/62
>75 years	38/83	49/88
Gender		
Male	31/63	35/73
Female	42/87	46/77
Hunt-Hess grade		
I-III	65/122	70/113
IV	8/28	11/37
Aneurysm size		
≤5mm	34/56	37/64
>5mm	39/94	44/86
Clot size		
Diffuse thick	27/72	31/67
Diffuse thin/local thick/local thin	45/78	50/83
Surgical procedure		
clipping	65/130	76/138
coiling	8/20	5/12

### The secondary endpoint-postoperative complications

**Figure 5:**
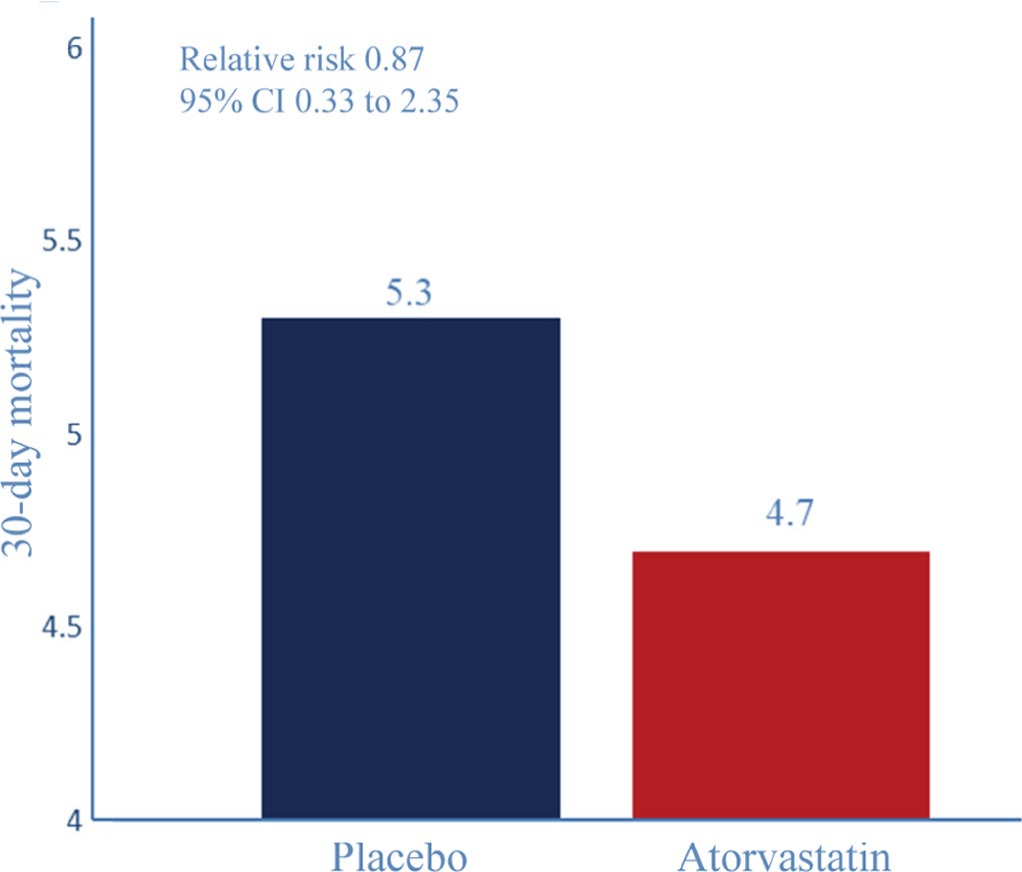
**30-day all-cause mortality.** Relative risk reduction percentages are rounded. Event rate (%) for 30-day all-cause mortality. RR 0.87, *P*=0.149, 95% CI 0.33–2.35.

According to the 30-day all-cause mortality, 7 (4.7%) of 150 patients in the atorvastatin group and 8 (5.3 %) of150 in the placebo group died within 30 days (RR 0.87, *P*=0.149, 95% CI 0.33–2.35, Figure 5).

**Figure 6:**
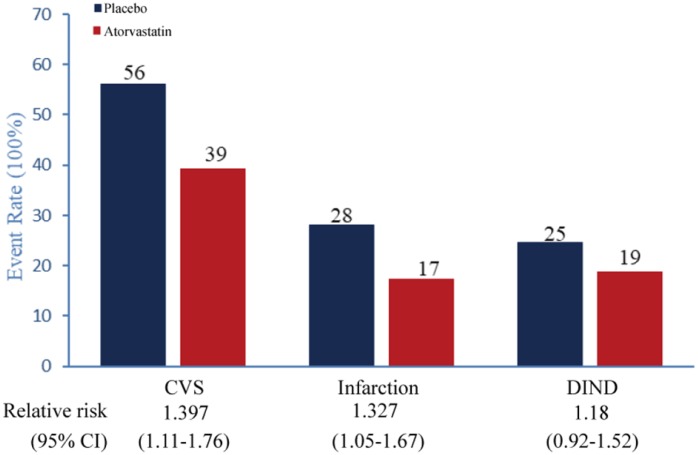
**Key secondary primary endpoints Event rate (%) for each of the individual components of the key secondary primary endpoint (all-treated, endpoint substituted; planned analysis).** DIND=delayed ischemic neurological deficit.

Event rates for each of the individual components of the primary composite endpoint are shown in Figure 6. The occurrence of postoperative CVS was significantly lower in the atorvastatin group than in the placebo group (Table 4, Figure 6, 39.3% vs 56%, *P* =0.004, Relative Risk 1.397, 95% CI 1.11 to 1.76). In this study, 27.3% (47/150) patients in the placebo group and 18.7% (28/150) in the atorvastatin group had delayed vasospasm-related new cerebral infarction (Table 4, Figure 6, *P*=0.027, Relative Risk 1.327, 95% CI 1.05 to 1.67). Even though group differences in the incidence of DIND were non-significant, the atorvastatin group had 6% lower risk of DIND incidence than the placebo group (Table 4, Figure 6, *P*=0.207, Relative Risk 1.18, 95% CI 0.92 to 1.52).

**Table 4:** Comparison of postoperative complications between the two groups.

**Variable**	**Placebo**	**Atorvastatin**	***P* value**
Number of patients	150	150	
Postoperative CVS	84(56%)	59(39.3%)	0.004*
Cerebral infarction	42(28%)	26(17.3%)	0.027*
DIND	37(24.7%)	28(18.7%)	0.207

Data are presented as numbers (%) and were compared between groups using the Pearson Chi-square test. Numeric variables were analyzed by use of an unpaired t test or Mann-Whitney *u* test.

* Indicates a statistically significant between groups difference (P < 0.05).

## DISCUSSION

20 mg/day atorvastatin for up to 14 days after aSAH operation had no significant effect on the primary endpoint of 6 month GOS or secondary endpoint of 30-day all-cause mortality. Subgroup analyses did not identify a subgroup of patients who might benefit from atorvastatin treatment. As most of the older patients in the study had Hunt-Hess grades I and II SAH, patients with Hunt-Hess grade V hemorrhages were excluded (by the protocol), the effect of atorvastatin in patients with poor Hunt-Hess grade or diffuse thick SAH cannot be determined. It was interesting that the incidence of postoperative CVS and cerebral infarction were reduced significantly in the atorvastatin treatment group relative to the placebo group. Lack of improvement might also have occurred if vasospasm contributed to DIND in the atorvastatin group. The findings may indicate that atorvastatin and nimodipine can enhance the effect of anti-CVS; atorvastatin is synergistic with nimodipine when combined.

Our trial has several strengths. The trial included many older patients, was masked, and more than 99% (297/300) of patients were followed up for assessment of a clinically relevant outcome. Atorvastatin treatment was well-tolerated, and no patients developed reversible side effects that required earlier cessation of atorvastatin treatment. All patients received TCD monitoring every day. In addition, most RCT or clinical retrospective studies have explored the effect of simvastatin, rosuvastatin, and pitavastatin on aSAH recently [10, 15, 16, 19–25]. The limitations of our study were as follows: 1. We collected key baseline and outcome data, but did not call patients back for detailed assessment of quality of life. 2. All of our patients were treated with injected nimodipine; whether combination of this vasodilator with atorvastatin contributed to adverse events needs further analysis. 3. Few patients with poor clinical condition (Hunt-Hess V) at admission were included, skewing our results. 4. Only 20 patients in the placebo group and 12 in the atorvastatin group received treatment by the coiling method, so we cannot evaluate the effect of atorvastatin on different treatments. 5. We used a single conventional dose of atorvastatin (20mg/day), which may be insufficient. 6. We did not include patients aged over 90 years or less than 60 years.

Most clinical trials testing medical treatments for prevention of vasospasm have been disappointing. Randomized trials assessing tirilazad, nicardipine, statins, magnesium, and haemodynamic manipulations have not shown consistent benefit [23]. Even though clazosentan significantly decreased angiographic vasospasm by blocking the actions of endothelin 1 in CONSCIOUS-1 trial, in CONSCIOUS-2 clazosentan had no significant effect on mortality, vasospasm-related morbidity, or functional outcome [5]. The statin treatment inefficiency observed may be associated with sample size, dose of statin, category of statin, and inclusion criteria by their analysis [5, 14, 17, 21–24]. Choi et al [25] reported that meta-analysis of 8 RCTs comprising 1150 patients indicated a significant reduction in DINDs and mortality in aSAH patients with high-dose statin usage (RR, 0.63; 95% CI, 0.42–0.95; P = 0.03; I2 = 0%; and RR, 0.36; 95% CI, 0.15–0.86; P = 0.02; I2 = 0%, respectively). Shen et al [26] also reported six RCTs and 2 prospective cohort studies included a total of 1461 patients, which demonstrated a significant decrease in the incidence of cerebral vasospasm (RR 0.76, 95% CI, 0.61–0.96) in patients treated with statins after aSAH. Unfortunately, both of two meta-analysis showed that no significant benefit was observed for mortality and functional outcomes [25, 26]. Older patients would have a good outcome as it's poor cerebral vascular may need statins to improve autoregulation. Our pre-experiment had a similar result; older patients had better outcomes than younger patients. Even though ischemic-related events were the main cause of death in both groups, hydrocephalus, infections, and brain edema also can impact the outcome and mortality, and the overall death rate was the same in both groups. In the end, although atorvastatin did not improve outcome or 30-day all-cause mortality, it may still have an effect on aSAH.

Our previous research in rabbits indicated that the neuroprotection from acute treatment with atorvastatin following aSAH is associated with enhancement of cerebral vessel autoregulation and inhibition of brain AQP4 expression [11, 12]. Similarly, many experiments showed that statins delay CVS, ameliorate EBI, and improve outcomes of patients after SAH [11, 12, 18–20, 27]. By contrast, other studies found that the benefits of statins on functional outcomes and delayed CVS are controversial, or even invalid [14, 20, 22, 23]. In the present study, we demonstrated that atorvastatin ameliorated CVS and postoperative cerebral infarction after SAH, had no significant effect on 30-day all-cause mortality, DIND, or functional outcome. However, we found the incidence of DIND and 30-day all-cause mortality all decreased in the atorvastatin group relative to placebo group; the lack of statistical significance may be due to lower incidence and small sample size.

In this present study, we found no benefit for clinical outcome after acute stage atorvastatin treatment. The effects of longer-term or larger dose (40mg/day or 80 mg/day) of atorvastatin remain unknown. The effects on long-term activities of daily living (ADL) and cognitive functions also were unclear.

We found that administration of atorvastatin after aSAH improves clinical outcome, 30-day all-cause mortality and DIND. Acute atorvastatin together with nimodipine therapy reduced CVS and postoperative cerebral infarction after aSAH in elderly Chinese patients. Further investigation of elderly patients undergoing endovascular coiling of ruptured aneurysms and different doses are needed to fully understand the potential usefulness of atorvastatin for patients with aSAH.

## MATERIALS AND METHODS

### Study design

We performed a randomized, parallel-arm placebo-controlled trial in 3 neurosurgical institutions in China (Wuxi Clinical College of Anhui Medical University-904th Hospital of Joint Logistic Support Force of PLA, Renmin Hospital of Wuhan University and Taizhou fourth people's hospital), between October 2014 and Oct 2017. The study was designed to assess the superiority of the intervention. The study protocol was approved by the Anhui Medical University affiliated Wuxi Clinical College Clinical Research Ethics Committees (2014-YXLL-001). The study protocol received Ethics Committee approval from all of the participating centers. We obtained written informed consent from patients whose competence was established by their accurate orientation for time, place, and person, and understanding of the recruiter’s description of the trial, or otherwise from their next of kin or their legal representative. The trial recruitment was decelerated after warnings from the Chinese Food and Drug Administration and the Department of Health of Wuxi in Oct 2014 that the highest approved dose of atorvastatin, ie, 20 mg, was associated with an elevated risk of muscle injury, hepatorenal and kidney dysfunction, or myopathy. The everyday clinical data also was monitored by Hospital Rational and Health drug use review committee. Safety data were reviewed, and it was decided that the study should continue.

Patients were randomly assigned (1:1) to receive 20 mg/day atorvastatin or placebo within 72 h of aSAH and after neurosurgical clipping or coiling (Figure 1). Atorvastatin or placebo were administered orally for up to 14 days after the aSAH. Final follow-up was 6 months after aSAH.

### Study patients

The inclusion criteria were as follows: radiological clear diagnosis of spontaneous subarachnoid hemorrhage; Hunt -Hess scale was 1 to 4; either gender; age 60-90 years; could be randomized within 72 h after the onset of SAH; an intracranial aneurysm that was considered to be the cause of the SAH and first onset. The exclusion criteria were as follows: patients unlikely to survive on admission; traumatic SAH; high cholesterol combined with diabetes; long-term use of atorvastatin as treatment group; abnormal liver enzymes, rhabdomyolysis or myopathy; history of mental illness or epilepsy; severe lung disease or multiple organ dysfunction; researchers believed patients were not suitable clinical subjects or participation in other clinical trials

### Randomization and masking

A permuted-block randomization was performed using a computer.system with an allocation list generated random numbers (in a 1:1 ratio) using SPSS 14.0 software (SPSS Institute, Hefei, Anhui Medical University) by a statistician not associated with the project team to protect the blinding and integrity of the study, and the results of randomization were sealed in sequentially numbered envelopes and stored at the site of investigation until the end of the study. During the study period, all inclusion patients were randomly assigned to receive either atorvastatin (20mg/day, 14 day) or placebo. A study nurse administered the study drugs according to the randomization sequence. Both the study members and the patients were blinded to the study drug allocation. The randomization envelopes were opaque. If an emergency such as severe hepatic failure occurred, then two experts could request unmasking of the treatment allocation, or adjust or interrupt the study drug if necessary; all situations were documented.

Patient demographics, medical history and relevant investigation results were collected. The severity of the SAH was scored clinically using the Hunt-Hess grading scale and radiologically using the Fisher scale.

### Standard of care

In the 3 participating centers, ruptured aneurysms were usually treated (clipping or coiling) within 24 hours after admission. Nimodipine was routinely started on admission and continued for 14 days in both groups. When clinical vasospasm developed, hypertensive treatment with an elevated mean arterial blood pressure of ≥20 mm Hg was started to maintain a relative higher perfusion. We adopted daily Transcranial Doppler (TCD) ultrasonography performed using a 2-MHz probe mounted on a specially designed head frame as a surrogate method to measure CVS. Computed tomographic perfusion (CTP) was performed in two of the participating centers for research purpose. Cerebral digital subtraction angiography (DSA) was not usually performed as it was available in only one hospital.

### Procedures

All patients underwent baseline cranial CT and CT angiography(CTA) or DSA. TCD ultrasonography was used to evaluate CVS daily. Cranial CT re-examination was obtained every 24–48 h after the aneurysm was clipped or coiled, for re-examination at discharge, and 6 months after aSAH. Neurological assessment was completed by two nurses every 2 h from initiation of study drug until day 14 using the Glasgow Coma Scale. If the patient had worsening of neurological symptoms, as defined previously [15], then CT, CTA, or DSA was required. At 6 months after randomization, patients were telephone interviewed to obtain GOS scores by a nurse or clinician with no knowledge of the treatment allocation.

All clinical and imaging data were assessed by a masked, independent diagnostic and assessment committee; this committee included two radiologists and two sonographers. Angiographic vasospasm and vasospasm-related new cerebral infarction were confirmed by radiologists. TCD vasospasm was confirmed by sonographers. A clinical assessment committee include one neurosurgeon, one neurointensivist, and one neurocritical care physician; all assessed the primary endpoint together.

The primary endpoint assessed GOS at 6 months after aSAH, dichotomized as good (≥4) or poor (<4) outcome. The secondary efficacy endpoint assessed all-cause mortality at 30 days after aSAH, CVS, vasospasm-related new infarction and DIND within 2 weeks post-aSAH. CVS, vasospasm-related new infarction, and DIND were defined previously [16] as follows: Angiographic vasospasm was defined as focal or generalized reduction of cerebral arterial caliber on conventional cerebral angiogram. TCD vasospasm was defined as any peak systolic middle cerebral artery velocity (PSV_MCA_) >200 cm/s and a Lindegaard ratio of >3. Vasospasm-related new infarction was defined where vasospasm was the relevant contributing factor or primary cause, and the development of a new lesion consistent with infarction on CT or MRI. DIND was defined as any 2 or more point fall in Glasgow Coma Scale or unaccountable new focal neurological deficit lasting ≥2 hours.

### Statistical analysis

On the basis of previous data we estimated that 212 patients would be required to confirm any effect with an α of 5% and 80% power [17]. For sample size estimation, we assumed that the atorvastain group had a 48% rate of good GOS compared with the placebo group with 46% based on our preliminary trial; 290 patients would be required (80% power and 2-sided,α=0.05, a 10% loss to follow-up). We decided to enroll 300 patients. A research nurse entered all baseline and outcome data in the study database; data were collected on handwritten forms and archived in a password-protected electronic database. Treatment effect was tested by logistic regression adjusted for Hunt-Hess grade (I, II, ≥III) with the Wald χ^2^ test used to determine treatment effect. We described the incidence and relative risk of dichotomous variables for the atorvastatin-treated group relative to the placebo group, with corresponding 95% CIs. The first exploratory end point was the GOS. For the GOS endpoint, if no GOS score was available, a score of 4 (lower moderate disability) was assigned when there was no clinical evidence of prior neurological impairment, and a score of 3 (lower severe disability) was assigned in any other situation when a patient was alive at 6 months. Demographics and safety data are reported as descriptive statistics (means, standard deviation) [5]. Categorical variables were analyzed with the χ^2^ test, continuity correction χ^2^ test or likelihood ratio χ^2^ test. Numeric variables were analyzed by use of an unpaired t test or Mann-Whitney u test. The difference (and 95% CI for the difference) between two medians was calculated with the Hodges-Lehmann estimator.

Statistical analyses were done on SPSS 14.0 software with two-tailed tests wherever appropriate and P values less than 0.05 were considered to be of statistical significance. The Clinical Research Ethics Committee from Anhui Medical University, Wuxi Clinical College (904^th^ Hospital of PLA) was involved in overseeing the data. The study is registered with http://www.chictr.org.cn, number ChiCTR-IPR-14005395.

### Ethics approval and consent to participate

The study protocol was approved by the Anhui Medical University affiliated Wuxi Clinical College Clinical Research Ethics Committee. We obtained written informed consent from the family members of patients whose competence was established by accurate orientation to time, place, and person and understanding of the recruiter’s description of the trial. Otherwise, consent was obtained from the patient’s next of kin or legal representative.

## Supplementary Material

Supplementary File 1
