# Supplementary material for: Atorvastatin reduces cerebral vasospasm and infarction after aneurysmal subarachnoid hemorrhage in elderly Chinese adults
Source: Aging (Albany NY). 2020 Feb 7;12(3):2939–51. doi: 10.18632/aging.102788 (PMC7041764; doi:10.18632/aging.102788)
Supplement: Supplementary File 1 [file aging-12-102788-s001..docx]

**Atorvastatin for treating spontaneous subarachnoid hemorrhage: study protocol for a randomized double-blind placebo-controlled trial**

**STUDY PROTOCOL**

# Jun-hui Chen*, Yu-hai Wang, Qian-xue Chen

**Abstract**

**Background:** Animal studies have confirmed that statins have neuroprotective effects during and following a subarachnoid hemor- rhage; however, the therapeutic effect of statins in humans remains controversial. The interpretation of data currently available on the clinical application of statins to spontaneous subarachnoid hemorrhage is limited by the small sample sizes used in the studies, making it difficult to draw valid conclusions regarding the multiple neuroprotective effects of statins. Thus, we propose to perform a random- ized double-blind placebo-controlled parallel-group clinical trial to determine the effects of atorvastatin on spontaneous subarachnoid hemorrhage, apoptosis-related factors, and serum inflammatory factors in cerebrospinal fluid and to observe its neuroprotective effect mediated by relieving vasospasm.

**Methods/Design:** This is a randomized parallel-group placebo-controlled double-blind clinical trial. This trial will recruit 300 patients with spontaneous subarachnoid hemorrhage from the Department of Neurosurgery, 101st Hospital of PLA (Wuxi Taihu Hospital), Renmin Hospital of Wuhan University. These patients will be equally and randomly assigned to atorvastatin (20 mg/day) and placebo control groups. Outcomes will be evaluated at baseline, 3, 5, and 14 days after hemorrhage, and 6 months after discharge. The primary endpoint assessed GOS at 6 months after aSAH, dichotomized as good (≥4) or poor (<4) outcome. The second primary efficacy endpoint assessed all-cause mortality at 30 days after aSAH, CVS, vasospasm-related new infarction and delayed ischaemic neurological deficit(DIND) due to vasospasm within 2 weeks post-aSAH.

**Discussion:** The results of this trial will provide data on the clinical application and neuroregenerative effect of atorvastatin in the acute stage of spontaneous subarachnoid hemorrhage.

**Trial registration:** This trial was registered with the Chinese Clinical Trial Registry (ChiCTR-IPR-14005395) on 18 May 2014.

**Key words:** clinical trial; atorvastatin; spontaneous subarachnoid hemorrhage; statins; randomized controlled trial

**Funding:** This study was supported by the Army Scientific Research Foundation of Nanjing Military Region of Chinese PLA; the Foundation of the 101 Hospital of Chinese PLA

**introduction**

Subarachnoid hemorrhage mainly refers to when a trau- matic or non-traumatic intracranial hemorrhage, caused by a variety of reasons, induces blood to flow into the subarachnoid space (Welty and Horner, 1990; King and Martin, 1994). Non-traumatic subarachnoid hemorrhage is also known as spontaneous subarachnoid hemorrhage. Blood gathered in the subarachnoid space can cause severe inflammation as well as an increase in nitric oxide, en- dothelin, oxyhemoglobin, and proinflammatory cytokine levels. The collected blood may also lead to vascular spasm, stenosis, and ischemic brain damage, resulting in early brain injury and cerebral vasospasm. Early brain injury and cerebral vasospasm are the main causes of neurological dysfunction and death after subarachnoid hemorrhage (Sobey and Faraci, 1998; Tseng et al., 2005; Rabinstein, 2011). The delayed neurological dysfunc- tion caused by spontaneous subarachnoid hemorrhage is not primarily caused by vascular spasm, but is possibly due to early vascular dysfunction, indicating that early administration of drugs for endothelial protection may be beneficial to the prognosis of patients. Cerda et al. (2015) verified that in addition to lowering lipid, statins improve endothelial function by reducing oxygen free radical production and increasing nitric oxide synthesis in endothelial cells. Statins resist platelet aggregation (Kato et al., 2004; Antoniades et al., 2011; Luzak et al., 2012) and antagonize coagulation factor VII activity (Ural and Avcu, 2006; Dietzen et al., 2007) by elevating nitric oxide synthase activity. Statins also improve overall fibrinolytic activity, reduce the inflammatory response, maintain the integrity of the blood-brain barrier, and ease cerebral vasospasm to mitigate subarachnoid hemorrhage (Cheng et al., 2009).

Many animal studies have shown that statins have neu- roprotective effects on subarachnoid hemorrhage (Tseng et al., 2005; Chou et al., 2008; McGirt et al., 2009; Ver- gouwen et al., 2009; Garg et al., 2013; Kirkpatrick et al., 2014; Yoshimura et al., 2014), but its clinical therapeutic effects remain controversial. Garg et al. (2013) confirmed that simvastatin may relieve cerebral vasospasm after sub- arachnoid hemorrhage, but cannot improve patient outcome. At present, there are few data concerning clinical applica- tion of statins in patients with spontaneous subarachnoid hemorrhage, and those studies that do exist have small sample sizes. To reach a convincing conclusion, the results of a large-sample, double-blind, randomized, placebo- controlled trial are needed. Thus, the present proposed ran- domized, parallel-group, placebo-controlled, double-blind clinical trial has been designed to determine the effects of atorvastatin on spontaneous subarachnoid hemorrhage,

apoptosis-related factors, and serum inflammatory factors in cerebrospinal fluid.

**Methods/design**

## Study design

This randomized, parallel-group, placebo-controlled, double-blind clinical trial will be conducted in the De- partment of Neurosurgery at 101st Hospital of PLA (Wuxi Taihu Hospital) and Renmin Hospital of Wuhan University.

## Ethical considerations and informed consent

An investigator or investigator-authorized personnel will explain the benefits and risks of participation to the study to each patient, family member, and guardian and obtain written informed consent. This project has obtained the written approval of the Medical Ethics Committee of 101st Hospital of PLA (Wuxi Taihu Hospital) (approval No. 2014-YXLL-001) and is in accordance with the guidelines of the *Declaration of Helsinki*, formulated by the World Medical Association.

## Study participants

Patients with spontaneous subarachnoid hemorrhage hospitalized in the Department of Neurosurgery at 101st Hospital of PLA and Renmin Hospital of Wuhan University will be recruited to participate in this study.

### *Inclusion criteria*

Patients meeting all of the following criteria will be con- sidered for admission to the trial:

- Aged 60–90 years; both sexes
- Sudden severe headache, vomiting, and meningeal ir-

ritation (Suarez, 2015)

- Abnormal computed tomography (CT) scans of the brain or a lumbar puncture showing that the cerebro- spinal fluid is bloody and under increased pressure (Smith et al., 2013; Suarez, 2015)
- First onset of symptoms
- Time of atorvastatin administration is within 72 hours after hemorrhage
- Informed consent of patients or their family members

### *Exclusion criteria*

Patients presenting with one of the following criteria will be excluded from the trial:

- Traumatic subarachnoid hemorrhage
- Hyperlipidemia combined with diabetes mellitus
- Abnormal liver enzymes, myopathy, and rhabdomyolysis
- History of mental illness or epilepsy
- Serious heart and lung disease or multiple organ dys- function
- Recent chemotherapy and radiotherapy
- Currently participating in other clinical trials

## Randomization

The 300 participants will be numbered according to their time of admission by a statistician using a table of random numbers. The participants will be equally assigned to either the atorvastatin group or a placebo control group. Doctors, patients, and assessors will be blinded regarding patient group assignments to avoid measurement bias.

## Blinding and emergency unblinding

The treatment program for each participant will be gener- ated using a random allocation sequence and placed in ordered, sealed, opaque envelopes. The blinded codes will be preserved by the drug administrator. If a patient has a serious adverse event and it is necessary to immediately determine the drug administered, a principal investigator of the research unit will open that patient’s envelope. Once

the data are unmasked, the patient will be discontinued from the trial. The clinical research associate will be informed of the result. Researchers will record the reason and date of the unmasking and sign the case report form.

## Interventions

Patients in both groups will receive conventional treat- ments, such as sedation, absolute bed rest, fluid balance, and hemostasis.

Atorvastatin group: in addition to the conventional treat- ments, patients will be orally administered atorvastatin (trade name: Lipitor; approval No. GYZZ H2005140; Pfizer Dalian Pharmaceutical Plant, Dalian, China), 20 mg for each day.

Placebo control group: in addition to the conventional treatments, patients will be orally administered placebo (starch tablets with the same appearance as atorvastatin). The dosage will be consistent with that for atorvastatin.

A flow chart for this clinical trial is shown in **Figure 1** and **Figure 2**.


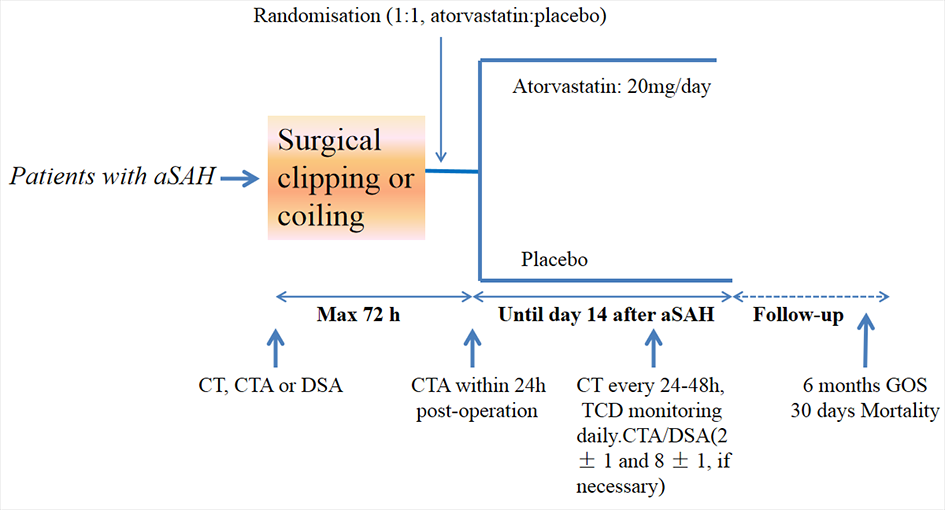


Figure 1


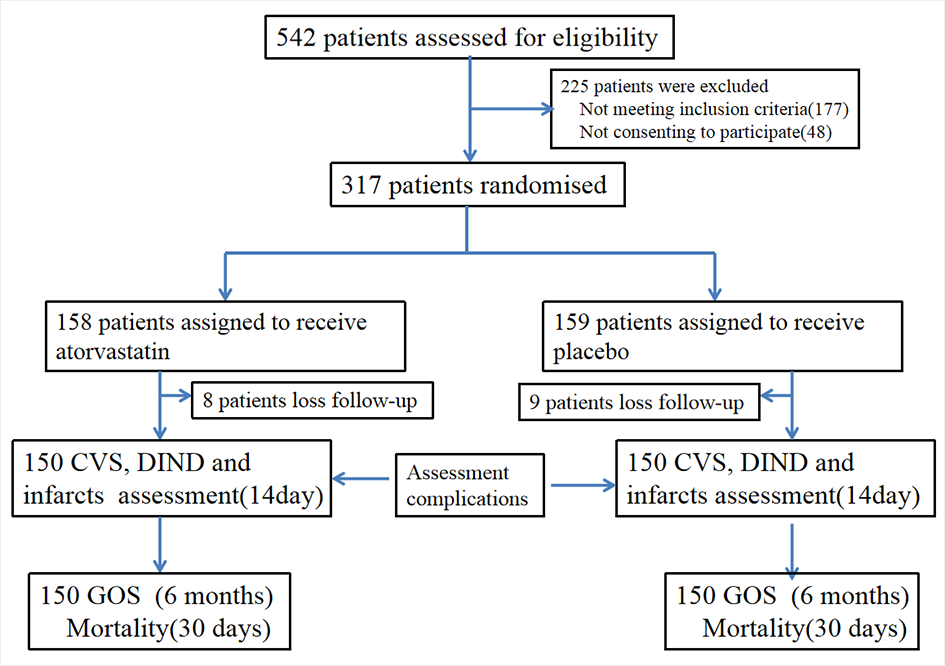


Figure 2

## Outcome measures

### *Primary outcomes*

- The primary endpoint assessed GOS at 6 months after aSAH, dichotomized as good (≥4) or poor (<4) outcome.

### *Secondary outcomes*

The second primary efficacy endpoint assessed all-cause mortality at 30 days after aSAH, CVS, vasospasm-related new infarction and delayed ischaemic neurological deficit(DIND) due to vasospasm within 2 weeks post-aSAH.

The defined of CVS, vasospasm-related new infarction and DIND can be consult at previously study as follows:

Angiographic vasospasm was defined as focal or generalized reduction of cerebral arterial caliber on conventional cerebral angiogram. TCD vasospasm was defined as any peak systolic middle cerebral artery velocity (PSVMCA) ＞200 cm/s and a Lindegaard ratio of ＞3. Vasospasm-related new infarction was defined that vasospasm was the relevant contributing factor or primary cause, and the development of a new lesion consistent with infarction on CT or MRI. DIND was defined as any 2 or more point fall in Glasgow Coma Scale or unaccountable new focal neurological deficit lasting ≥2 hours.

## Follow-up

The participants will be followed up 6 months after discharge. The patients will be requested by telephone to return to the hospital for examinations that will include CT angiography plus CT perfusion imaging, conventional CT, blood testing, cerebrospinal fluid analysis, transcranial Doppler ultrasonography, and neurological scale assessments. If a patient is lost to follow-up, the last values assessed will be considered the values for the 6-month follow-up.

## Safety

Adverse reactions may include gastrointestinal dysfunction, immune system dysfunction, insomnia, forgetfulness, nervous system dysfunction, pruritus, myalgia, arthralgia, and serum transaminase and phosphocreatine kinase abnormalities.

To facilitate truthful reporting by patients of the changes in their conditions after drug use, the doctors will avoid asking patients leading questions. Adverse events or un- anticipated side effects (symptoms, signs, and laboratory tests) will be recorded in addition to the curative effects.

If severe adverse events occur, researchers will immediately take appropriate therapeutic measures. The drug will be withdrawn. Drug withdrawal time, adverse events, symptoms, occurrence time, duration, treatment, and out- come will be recorded. The investigators will report all severe adverse events to the responsible unit, reporting units, the ethics committee, the Drug Supervision and Ad- ministration Department, and the relevant administrative departments within 24 hours. The investigators will sign and date the report.

## Data management

The medical records will be electronically saved on a computer. The accuracy of data recorded on the case data form will be verified. Any changes or corrections will retain a clear record of the original. The investigator will record the date of changes or corrections, sign his or her name, and explain the reasons for the change (if necessary). The medical records along with any changes, deletions, or additions will be electronically saved on a computer. The audit trail will be set so that it is protected by the system and will not be able to be modified or edited by anyone. The clinical data management system will have authority management such that only authorized personnel will be allowed to operate it. The use of an electronic signature is a common means of managing electronic management systems. Only authorized personnel can log onto the system for data entry or modification or to view the data, preventing unauthorized logins and access to any clinical data so that it remains secure and confidential. In accordance with the China Drug Clinical Trial Management Specification, all records will be preserved for at least 5 years after completion of the trial.

## Statistical analysis

For sample size estimation, we assumed that the Atorvastain group had a 48% rate of good GOS compared with the placebo group with 46% based on our preliminary trial, that 290 patients would be required (80% power and 2-sided,α=0.05, a 10% loss to follow-up). We decided to enroll 300 patients. A research nurse entered all baseline and outcome data in the study database, data were collected on handwritten forms and archived in a password-protected electronic database. Treatment effect was tested by logistic regression adjusted for Hunt-Hess grade (I, II, ≥III) with the Wald χ2 test used to determine treatment effect. We described the incidence and relative risk reduction of dichotomous variables for the atorvastatin-treated group relative to the placebo group, with corresponding 95% CIs. The first exploratory end point was the GOS. For the GOS endpoint, if no GOS score was available, a score of 4 (lower moderate disability) was assigned when there was no clinical evidence of prior neurological impairment, and a score of 3 (lower severe disability) assigned in any other situation when a patient was alive at 6 months. Demographics and safety data are reported as descriptive statistics (means, standard deviation). Categorical variables were analyzed with the χ2 test, continuity correction χ2 test or likelihood ratio χ2 test. Numeric variables were analyzed by use of an unpaired t test or Mann-Whitney u test. The difference (and 95% CI for the difference) between two medians was calculated with the Hodges-Lehmann estimator. As our previous study and related research had confirmed that Hunt-Hess, Age, Aneurysm size, and Clot size impact outcome. So, We want to know that it would also be interesting to know whether treatment measures work in certain subgroups in this trial. We revised and add the related message in this manuscript. Thank you again for your advice.

We analyzed outcome data and safety in the intention-to-treat population. We also did per-protocol analysis for the primary endpoint. We did not do an interim analysis. Statistical analyses were done on SPSS 14.0 software with two-tailed tests wherever appropriate and P values less than 0.05 were considered to be of statistical significance. The Clinical Research Ethics Committee from Anhui Medical University, Wuxi Clinical College (904th Hospital of PLA) was involved in overseeing the data. The study is registered with www.chictr.org.cn, number ChiCTR-IPR-14005395.

## Trial quality assurance and control

JHC, one of the authors, will be responsible for quality assurance and control. A clinical research associate as- signed by the sponsor will visit the hospital regularly to ensure that the protocol has been strictly followed and that all data are correctly recorded. The researchers will implement standard operating procedures for clinical trials before, during, and after the trial. During the trial, inspectors will monitor the correctness and integrity of the data using case registration forms. Researchers will be provided with training to enable uniform recording methods and judgment standards. Researchers will carefully record all information on case report forms to ensure that the data are correct and reliable. The investigators will use normal reference ranges as standards to determine abnormal laboratory test results. All observations and findings will be checked to ensure data reliability and to ensure that the conclusions come from the original data. In the clinical trials and data processing stage, there will be corresponding data management measures. Active measures will be taken to control the dropout rate to within 20%.

**discussion**

Sedation, absolute bed rest, fluid balance, and hemostasis are for the symptomatic treatment of patients with spontaneous subarachnoid hemorrhage. Such conventional treatments cannot effectively restore the patient’s neurological

condition. Thus, the goal of this study is to confirm the multiple neuroprotective effects of atorvastatin, including reducing inflammation, relieving vasospasm, improving endothelial cell function, and confronting blood coagula- tion activity in the treatment of spontaneous subarachnoid hemorrhage through this clinical trial. It remains poorly understood whether the addition of atorvastatin to the treatment protocol will improve outcome in the recovery of neurological function. The most common adverse reaction of atorvastatin is myopathy, which resolves after drug withdrawal because it occurs with atorvastatin administra- tion in a dose-dependent manner (Hermann et al., 2006; Soininen et al., 2006; Abdelbaset et al., 2014). The effect of atorvastatin on liver function is limited (Gershovich and Lyman, 2004; Tikkanen et al., 2013; Kalantari and Naghipour, 2014). Therefore, atorvastatin is expected to become a relatively safe method for the treatment of spontaneous subarachnoid hemorrhage. If this large sample size, ran- domized, parallel-group, placebo-controlled, double-blind clinical trial verifies the effectiveness of atorvastatin, this drug will be considered effective for the treatment of spontaneous subarachnoid hemorrhage.

## Trial status

Ongoing and recruiting at the time of submission.

**References**

Abdelbaset M, Safar MM, Mahmoud SS, Negm SA, Agha AM (2014) Red yeast rice and coenzyme Q10 as safe alternatives to surmount atorvastatin-induced myopathy in hyperlipidemic rats. Can J Physiol Pharmacol 92:481-489.

Antoniades C, Bakogiannis C, Leeson P, Guzik TJ, Zhang M-H, Tou- soulis D, Antonopoulos AS, Demosthenous M, Marinou K, Hale A, Paschalis A, Psarros C, Triantafyllou C, Bendall J, Casadei B, Stefanadis C, Channon KM (2011) Rapid, direct effects of statin treatment on arterial redox state and nitric oxide bioavailability in human atherosclerosis via tetrahydrobiopterin-mediated endothe- lial nitric oxide synthase coupling. Circulation 124:335-345.

Cerda A, Fajardo CM, Basso RG, Hirata MH, Hirata RD (2015) Role of microRNAs 221/222 on statin induced nitric oxide re- lease in human endothelial cells. Arq Bras Cardiol 104:195-200.

Cheng G, Wei L, Zhi-dan S, Shi-guang Z, Xiang-zhen L (2009) Atorvastatin ameliorates cerebral vasospasm and early brain inju- ry after subarachnoid hemorrhage and inhibits caspase-dependent apoptosis pathway. BMC Neurosci 10:7.

Chou SH, Smith EE, Badjatia N, Nogueira RG, Sims JR, Ogilvy CS, Rordorf GA, Ayata C (2008) A randomized, double-blind, placebo-controlled pilot study of simvastatin in aneurysmal sub- arachnoid hemorrhage. Stroke 39:2891-2893.

Dietzen DJ, Page KL, Tetzloff TA, Bohrer A, Turk J (2007) Inhi- bition of 3-hydroxy-3-methylglutaryl coenzyme A (HMG CoA) reductase blunts factor VIIa/tissue factor and prothrombinase activities via effects on membrane phosphatidylserine. Atertio Thromb Vasc Biol 27:690-696.

Ehrlich G, Kirschning T, Wenz H, Hegewald AA, Groden C, Schmiedek P, Scharf J, Seiz-Rosenhagen M (2016) Is there an influence of routine daily transcranial doppler examination on clinical outcome in patients after aneurysmal subarachnoid hem- orrhage? World Neurosurg doi: 10.1016/j.wneu.2015.11.091.

Garg K, Sinha S, Kale SS, Chandra PS, Suri A, Singh MM, Kumar R, Sharma MS, Pandey RM, Sharma BS, Mahapatra AK (2013) Role of simvastatin in prevention of vasospasm and improving functional outcome after aneurysmal subarachnoid hemorrhage: a prospective, randomized, double-blind, placebo-controlled pilot trial. Br J Neurosurg 27:181-186.

Gershovich OE, Lyman AE (2004) Liver mitochondrial respiratory function and coenzyme Q content in rats on a hypercholesterol- emic diet treated with atorvastatin.Pharmacotherapy 24:150-154.

Gocan S, Fisher A (2008) Neurological assessment by nurses using the National Institutes of Health Stroke Scale: implementation of

best practice guidelines. Can J Neurosci Nurs 30:31-42.

Hermann M, Bogsrud MP, Molden E, Åsberg A, Mohebi BU, Ose L, Retterstøl K (2006) Exposure of atorvastatin is unchanged but lactone and acid metabolites are increased several-fold in pa- tients with atorvastatin-induced myopathy. Clin Pharmacol Ther 79:532-539.

Kalantari S, Naghipour M (2014) Statin therapy and hepatotoxicity: Appraisal of the safety profile of atorvastatin in hyperlipidemic patients. Adv Biomed Res 3:168.

Kato T, Hashikabe H, Iwata C, Akimoto K, Hattori Y (2004) Statin blocks Rho/Rho-kinase signalling and disrupts the actin cytoskel- eton: relationship to enhancement of LPS-mediated nitric oxide synthesis in vascular smooth muscle cells. Biochim Biophys Acta 1689:267-272.

King WA, Martin NA (1994) Critical care of patients with subarach- noid hemorrhage. Neurosurg Clin N Am 5:767-787.

Kirkpatrick PJ, Turner CL, Smith C, Hutchinson PJ, Murray GD, STASH Collaborators (2014) Simvastatin in aneurysmal sub- arachnoid haemorrhage (STASH): a multicentre randomised phase 3 trial. Lancet Neurol 13:666-675.

Luzak B, Rywaniak J, Stanczyk L, Watala C (2012) Pravastatin and simvastatin improves acetylsalicylic acid-mediated in vitro blood platelet inhibition. Eur J Clin Invest 42:864-872.

McGirt MJ, Garces Ambrossi GL, Huang J, Tamargo RJ (2009) Simvastatin for the prevention of symptomatic cerebral vaso- spasm following aneurysmal subarachnoid hemorrhage: a single- institution prospective cohort study. J Neurosurg 110:968-974

Nina P, Schisano G, Chiappetta F, Luisa Papa M, Maddaloni E, Brunori A, Capasso F, Corpetti MG, Demurtas F (2001) A study of blood coagulation and fibrinolytic system in spontaneous sub- arachnoid hemorrhage. Correlation with hunt-hess grade and out- come. Surg Neurol 55:197-203.

Okamura K (2014) Glasgow Coma Scale flow chart: a beginner's guide. Br J Nurs 23:1068-1073.

Rabinstein AA (2011) Secondary brain injury after aneurysmal sub- arachnoid haemorrhage: more than vasospasm. Lancet Neurol 10:593-595.

Smith A, Wu AHB, Lynch KL, Ko N, Grenache DG (2013) Multi- wavelength spectrophotometric analysis for detection of xantho- chromia in cerebrospinal fluid and accuracy for the diagnosis of subarachnoid hemorrhage. Clin Chim Acta 424:231-236.

Sobey CG, Faraci FM (1998) Subarachnoid haemorrhage: what happens to the cerebral arteries? Clin Exp Pharmacol Physiol 25: 867-876.

Soininen K, Niemi M, Kilkki E, Strandberg T, Kivist KT (2006) Muscle symptoms associated with statins: a series of twenty pa- tients. Basic Clin Pharmacol Toxicol 98:51-54.

Suarez JI (2015) Diagnosis and management of subarachnoid hem- orrhage. Continuum (Minneap Minn) 21:1263-1287.

Tikkanen MJ, Fayyad R, Faergeman O, Olsson AG, Wun CC, Las- key R, Kastelein JJ, Holme I, Pedersen TR (2013) Effect of inten- sive lipid lowering with atorvastatin on cardiovascular outcomes in coronary heart disease patients with mild-to-moderate base- line elevations in alanine aminotransferase levels. Int J Cardiol 168:3846-3852.

Tseng MY, Czosnyka M, Richards H, Pickard JD, Kirkpatrick PJ (2005) Effects of acute treatment with pravastatin on cerebral vasospasm, autoregulation, and delayed ischemic deficits after aneurysmal subarachnoid hemorrhage: a phase II randomized placebo-controlled trial. Stroke 36:1627-1632.

Ural AU, Avcu F (2006) HMG-CoA reductase inhibitors may affect thrombin generation by reducing factor VII activity in hyperlipid- emic patients. Thromb Res 118:665-666.

Vergouwen MD, Meijers JC, Geskus RB, Coert BA, Horn J, Stroes ES, van der Poll T, Vermeulen M, Roos YB (2009) Biologic ef- fects of simvastatin in patients with aneurysmal subarachnoid hemorrhage: a double-blind, placebo-controlled randomized trial. J Cereb Blood Flow Metab 29:1444-1453.

Welty TE, Horner TG (1990) Pathophysiology and treatment of sub- arachnoid hemorrhage. Clin Pharm 9:35-39.

Wilson JT, Pettigrew LE, Teasdale GM (1998) Structured interviews for the Glasgow Outcome Scale and the extended Glasgow Out- come Scale: guidelines for their use. J Neurotrauma 15:573-585. Yoshimura Y, Murakami Y, Saitoh M, Yokoi T, Aoki T, Miura K, Ueshima H, Nozaki K, SSS Research Group (2014) Statin use and risk of cerebral aneurysm rupture: a hospital-based case-control

study in Japan. J Stroke Cerebrovasc Dis 23:343-348.
